# Expression of concern: A study on the luminescence properties of gamma-ray-irradiated white light emitting Ca_2_Al_2_SiO_7_:Dy^3+^ phosphors fabricated using a combustion-assisted method

**DOI:** 10.1039/c9ra90023c

**Published:** 2019-03-27

**Authors:** Andrew Shore

**Affiliations:** Royal Society of Chemistry Thomas Graham House, Science Park, Milton Road Cambridge UK CB4 0WF advances-rsc@rsc.org

## Abstract

Expression of concern for ‘A study on the luminescence properties of gamma-ray-irradiated white light emitting Ca_2_Al_2_SiO_7_:Dy^3+^ phosphors fabricated using a combustion-assisted method’ by Geetanjali Tiwari *et al.*, *RSC Adv.*, 2016, **6**, 49317–49327.

*RSC Advances* is publishing this expression of concern in order to alert our readers that we are presently unsure of the reliability of the data presented and conclusions reported in the article.

The Royal Society of Chemistry was contacted by a reader who raised concerns about the reliability of the data presented in the paper. The authors have rechecked their data and repeated experiments and have found errors with the data reported in Fig. 10(a, b), 11(a, b), 12(a, b), 15, 18 and 19, and the method of calculation of the activation energy (Table 2).

The authors have provided replacement data and figures for consideration and say that the new data does not affect the conclusions of the paper. The Royal Society of Chemistry has asked the affiliated institution (Pt. Ravishankar Shukla University, India) to investigate this matter and establish whether the replacement figures provided by the authors provide an accurate representation of the experiments that were conducted, and confirm the integrity and reliability of the new data and figures provided. An expression of concern will continue to be associated with this manuscript until we receive information from the institution on this matter.

Andrew Shore

18th March 2019

Executive Editor, *RSC Advances*

## Supplementary Material

