# Correction and removal of expression of concern: A study on the luminescence properties of gamma-ray-irradiated white light emitting Ca_2_Al_2_SiO_7_:Dy^3+^ phosphors fabricated using a combustion-assisted method

**DOI:** 10.1039/d0ra90011g

**Published:** 2020-02-03

**Authors:** Geetanjali Tiwari, Nameeta Brahme, Ravi Sharma, D. P. Bisen, Sanjay K. Sao, S. J. Dhoble

**Affiliations:** School of Studies in Physics and Astrophysics, Pt. Ravishankar Shukla University Raipur C. G. India geetanjali.tiwari10@gmail.com namitabrahme@gmail.com; Department of Physics, Govt. Arts and Commerce Girls College Devendra Nagar Raipur C. G. India; Department of Physics, RTM University Nagpur Maharashtra India

## Abstract

Correction and removal of expression of concern for ‘A study on the luminescence properties of gamma-ray-irradiated white light emitting Ca_2_Al_2_SiO_7_:Dy^3+^ phosphors fabricated using a combustion-assisted method’ by Geetanjali Tiwari *et al.*, *RSC Adv.*, 2016, **6**, 49317–49327.

The authors wish to draw the reader’s attention to their closely related paper in *Optical Materials*,^1^ which was undergoing peer review at the same time as this *RSC Advances* paper. Ref. 1 was published shortly after this paper but should have been cited in this *RSC Advances* article.

The authors regret that the results in some of the figures and the conclusion part in the original manuscript were not correct. The corrected Fig. 10(a, b), 11(a, b), 12(a, b), 15, 18 and 19 are given below. In addition, the authors have recalculated the activation energy using a peak shape method, corrected Table 2 and corrected part of the conclusion section. These corrections do not affect the original conclusions of this paper.

The accuracy and integrity of the new data has been confirmed by the affiliated institution (Pt. Ravishankar Shukla University, India). Their enquiry concluded that “the data and figures provided by the corresponding authors in the correction notice are accurate representations of the experiments. The corrected figures, calculation of activation energy by peak shape method and the correction conclusion part do not affect the original conclusions of this paper. According to the above conclusions, the data and figures provided in this correction notice maintain the accuracy and integrity of the experiments.”

The new data and figures have also been reviewed by an independent expert and are provided below in order to fulfil the journal’s responsibility to correct the scientific record, in accordance with the guidelines provided by the Committee on Publication Ethics.

**Fig. 1:**
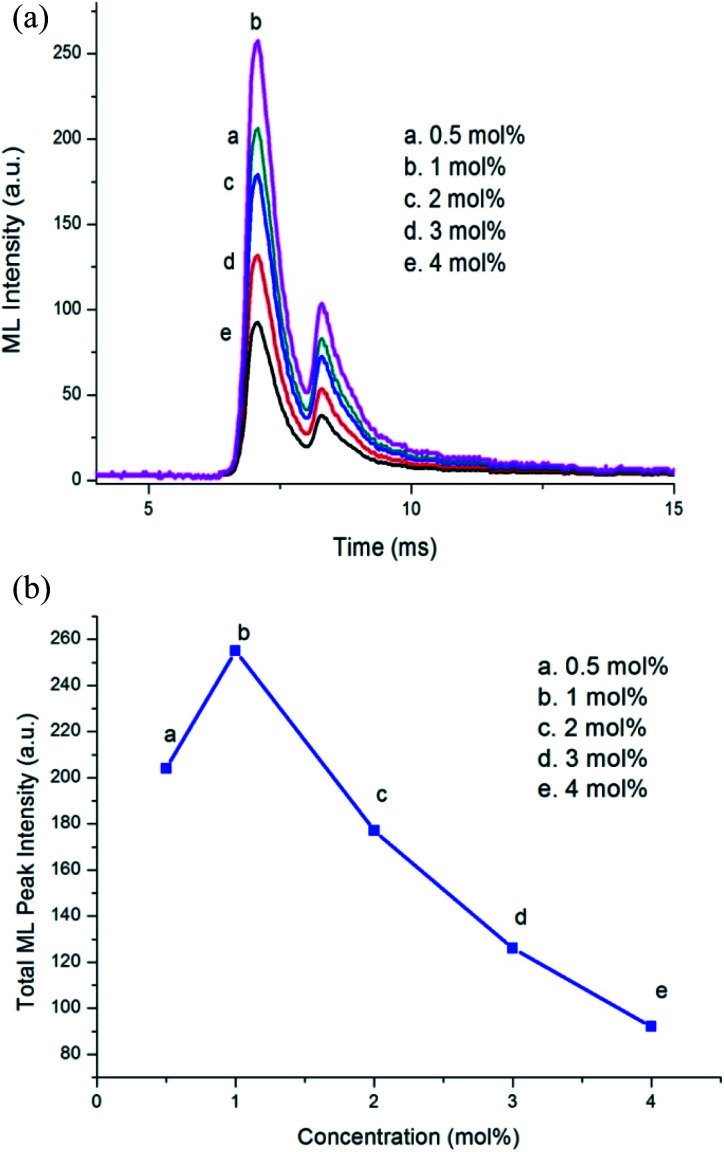
(a) ML intensity *versus* time curve for different Dy^3+^ concentrations. (b) Variation in ML peak intensity with Dy^3+^ concentration variation.

**Fig. 2:**
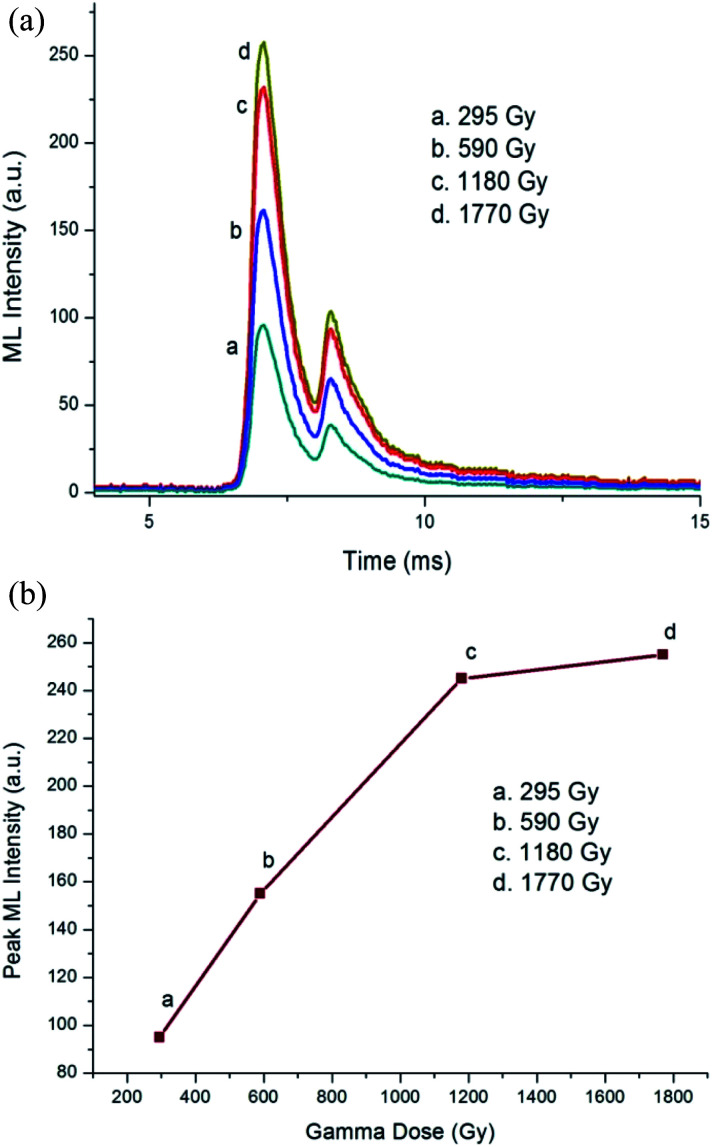
(a) ML intensity *versus* time curve for the γ-irradiated Ca_1.99_A_l2_SiO_7_:0.01Dy^3+^ phosphor. (b) Dependence of peak ML intensity on the γ-dose for the Ca_1.99_A_l2_SiO_7_:0.01Dy^3+^ phosphor.

**Fig. 3:**
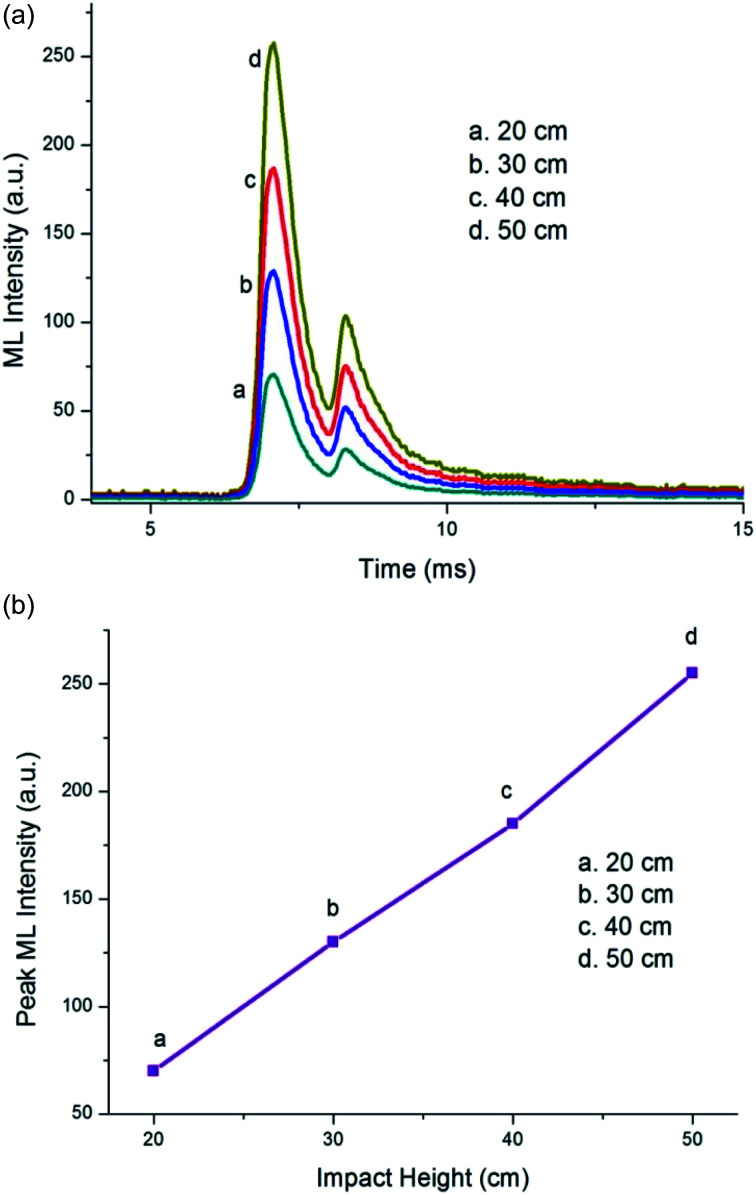
(a) Change in the ML intensity with impact height for the Ca_1.99_A_l2_SiO_7_:0.01Dy^3+^ phosphor after a 1180 Gy dose of γ-irradiation. (b) Peak ML intensity *versus* impact height for the Ca_1.99_A_l2_SiO_7_:0.01Dy^3+^ phosphor after a 1180 Gy γ-dose.

**Fig. 4:**
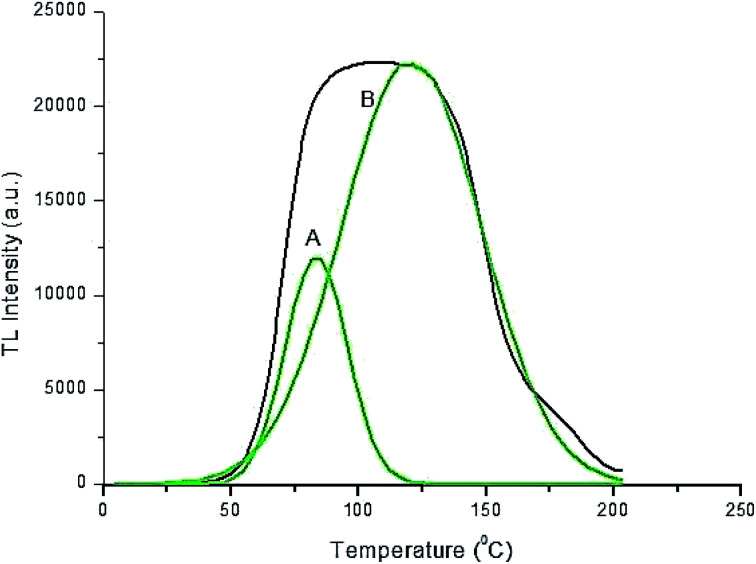
Peak deconvolution of TL glow curve of the Ca_1.99_A_l2_SiO_7_:0.01Dy^3+^ phosphor for a 1180 Gy γ-dose.

**Fig. 5:**
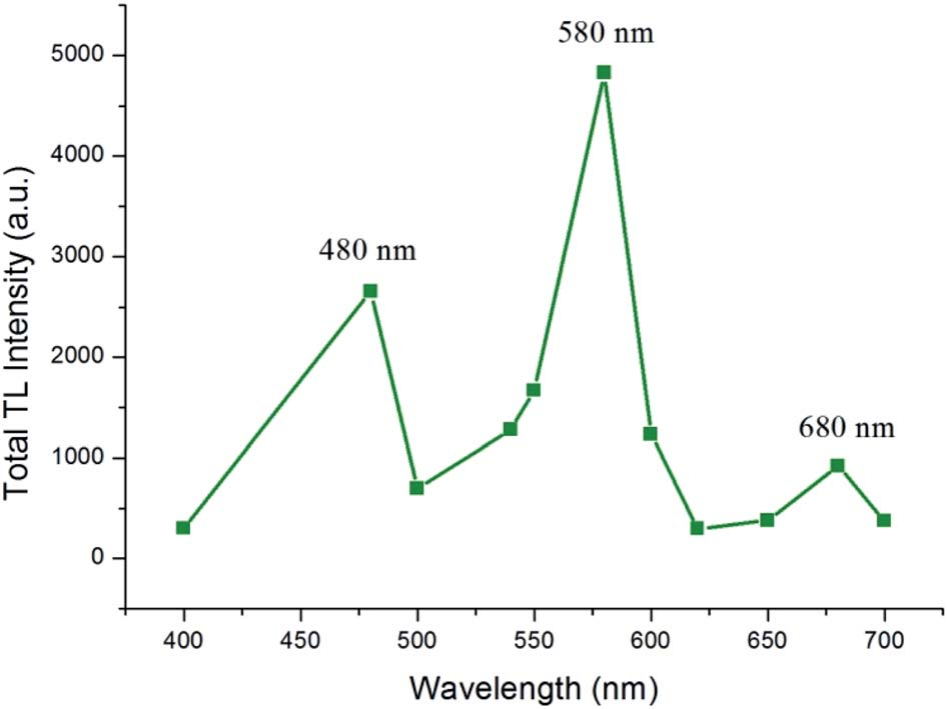
TL emission spectra of the Ca_1.99_A_l2_SiO_7_:0.01Dy^3+^ phosphor for a γ-dose of 1180 Gy.

**Fig. 6:**
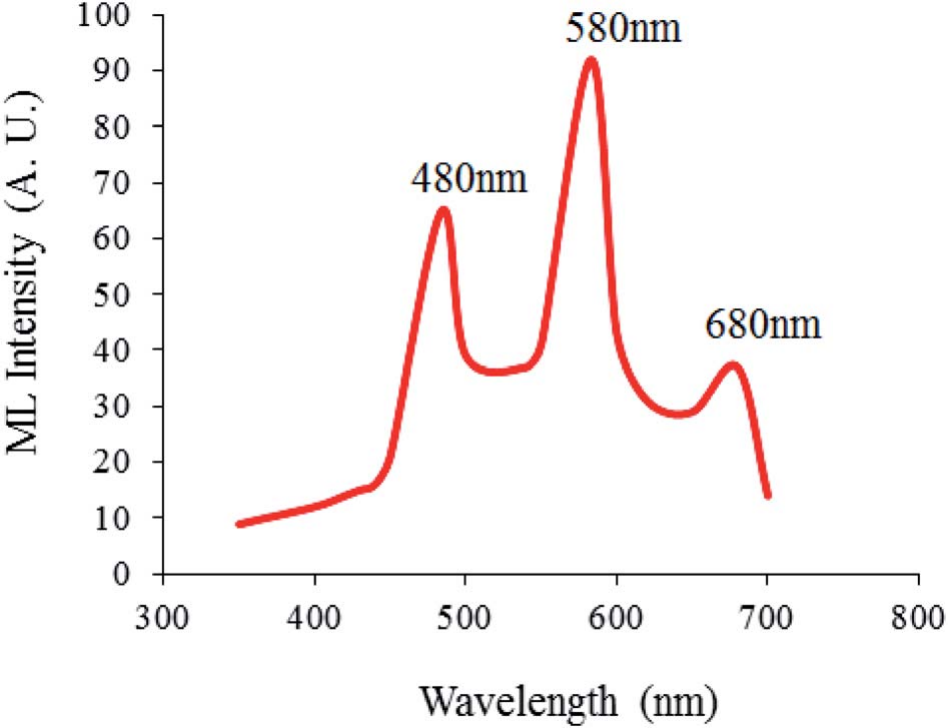
ML emission spectra of the Ca_1.99_A_l2_SiO_7_:0.01Dy^3+^ phosphor for a γ-dose of 1180 Gy.

The following portions of text concerning the discussion of Fig. 15, 18 and 19 have been updated to reflect the corrected figures.

Page 49324 (left column), paragraph 3: “The peak deconvolution of the TL curve is shown in Fig. 15. It has two dominant bands peaking at approximately 83.7 °C and 120.7 °C.”

Page 49325 (left column), paragraph 2: “Fig. 18 shows that the TL emission spectra of the Ca_2_Al_2_SiO_7_:Dy^3+^ phosphor exhibits a broad emission band centered on 480 (blue), 580 (yellow), and 680 (red)…”

Page 49325 (left column), paragraph 3: “Fig. 19 shows that the ML emission spectra of Ca_2_Al_2_SiO_7_:Dy^3+^ exhibits broad emission bands centered on 480 (blue), 580 (yellow) and 680 (red)…”

In the original article, the authors used the initial rise method to calculate the activation energy (see pages 49324–49325). The authors have now used the peak shape method to calculate the activation energy. As a result, Fig. 16 and 17 in the original article are now redundant and should be removed from the article. Details about the peak shape method used and a corrected Table 2 are provided here.

**“Peak shape method for calculation of activation energy.** Fig. 14(a) shows that the TL intensity starts to become saturated at 1180 Gy of γ-irradiation. The thermal activation energy *E* for the sample with 1180 Gy of γ-irradiation (associated with the trap depth) was calculated from the glow peak parameters using the following equation:*E* = 2*kT*_m_(1.26*T*_m_/*ω* − 1)where *ω* = *τ* + *δ* is the total half width intensity, *τ* is the half width at the low temperature side of the peak (*τ* = *T*_m_ − *T*_1_), *δ* is the half width towards the fall-off side of the glow peak (*δ* = *T*_2_ − *T*_m_), where *T*_1_ and *T*_2_ are the half-intensity temperatures on the low and high temperature side of the peak, respectively. *T*_m_ is the peak temperature at the maximum. The *μ* = *δ*/*ω* is a shape factor which differentiates between first and second order TL glow peak. The frequency factor was calculated by the formula *s* = [2*β*(1.26*T*_m_/*ω* − 1)/(*e*^2^*T*_m_)]exp(2.52*T*_m_/*ω*) where *β* is the (constant) heating rate and *T*_m_ is the maximum temperature. The TL parameters, *i.e.* the activation energy (*E*) and frequency factor (s^−1^) for the prominent glow peaks of the prepared phosphor are shown in Table 2.”

**Table 1:** Activation energy (*E*) and frequency factor (s^−1^) for the γ-irradiated Ca_2_Al_2_SiO_7_:Dy^3+^ phosphor

γ-Dose (Gy)	Heating rate (°C s^−1^)	*T*_m_ (K)	*T*_1_ (K)	*T*_2_ (K)	*τ* (K)	*δ* (K)	*ω* (K)	*μ* = *δ*/*ω*	Activation energy *E* (eV)	Frequency factor (s^−1^)
1180 (1^st^ peak)	5	356.7	341.29	370.9	15.47	14.17	29.64	0.47	0.54	1.24 × 10^11^
1180 (2^nd^ peak)	5	393.7	363.2	426.3	30.5	32.6	63.1	0.51	0.96	2.1 × 10^11^

The second half of the conclusion section, from “The TL/ML intensity increased with the increase…” until “…compared to solid state reaction methods”, should be changed to the following.

“The TL and ML intensity increased with a higher γ-dose indicating an increase in the concentration of traps with γ-dose. Te TL and ML intensity of the phosphor became saturated with a 1180 Gy gamma dose. The ML intensity increased approximately linearly with increasing impact velocity. The reason for the mechanoluminescence in the present phosphor was explained by a piezo-electrification model. Since the ML intensity of the γ-irradiated Ca_2_A_l2_SiO_7_:Dy^3+^ phosphor increased linearly, this phosphor may be used as a stress sensor. The chromaticity coordinates of a long afterglow spectra were measured and found a white afterglow of the phosphor. In this work Ca_2_A_l2_SiO_7_:Dy^3+^ phosphors were prepared by a combustion-assisted method for the first time to the best of our knowledge. The TL and ML intensities of the phosphor were measured after γ-irradiation. The result may be useful to detect and measure the γ-dose and to develop a γ-radiation sensor.”

This correction supersedes the information provided in the Expression of Concern related to this article.

The Royal Society of Chemistry apologises for these errors and any consequent inconvenience to authors and readers.

## Supplementary Material
